# When measurement outpaces meaning: Bioimpedance in Organ-on-Chip systems

**DOI:** 10.2478/joeb-2026-0001

**Published:** 2026-01-07

**Authors:** Sebastian Wegner

**Affiliations:** Sciospec Scientific Instruments GmbH,Germany

Organ-on-Chip (OoC) systems have long matured beyond proof-of-concept. They are no longer competing with animal models for acceptance, but on robustness of interpretation and comparability. Currently, optical and biochemical endpoint measurements dominate the analytical toolset. In contrast to these, bioimpedance offers something different: continuous, non-destructive observation, and access to dynamic biological questions. But without robust, repeatable and physically grounded interpretation, impedance is bound to remain a secondary QC signal rather than primary biological evidence. The question for the bioimpedance community is: What has to change for impedance to become more than a secondary signal? Crucially, this is not challenging bioimpedance theory, but is a reflection on its application in regimes where previously implicit assumptions must be made explicit. At its core, it is therefore not a question of measurement capability, but of whether physically qualified interpretation is treated as first-class research objective rather than downstream convenience.

## Misleading maturity perception is a trap

This interpretive gap is easily blurred by the apparent technical maturity of current OoC impedance platforms. Commercially available systems are already automated, scalable, and technically mature. In contrast, the interpretation layer required to translate signals into qualified biological information remains underdeveloped [1]. This mismatch creates a systematic risk: high-throughput impedance measurements generate large volumes of data whose sources of variance often cannot be decomposed into biological, geometric, or instrumental contributions [2]. Impedance changes attributed to biological responses are frequently distorted by system-level effects such as material sorption in widely used prototyping platforms like PDMS [3]. The result is not merely low-value data, but the systematic accumulation of datasets that appear rich, yet cannot be reproduced, compared, or elevated into evidence beyond their original setting. With measurement capability at hand but lacking interpretation clarity, the lack of standardization becomes a pressure point – but potentially also a distraction.

## Standardization must be rooted in physics

The lack of comparability in OoC impedance data is often framed as a reporting problem, exemplified by ongoing debates over whether results should be expressed in raw ohms or normalized Ω·cm^2^ [4]. In recent years, the community has made tangible progress through reporting guidelines [5] and standardization roadmaps [6], improving transparency and exposing hidden assumptions. However, reporting standards alone cannot resolve the underlying comparability problem. In miniaturized OoC systems, electric fields are highly non-uniform, and sensing volumes are strongly geometry-dependent [3]. Under these conditions, unit normalization without geometric correction risks creating a false sense of standardization. The central issue is therefore not how impedance values are reported, but whether the physical meaning of the measurement is conserved across platforms before metrics are compared.

Historical precedent reinforces this concern. Earlier phases of bioimpedance research became fragmented not because of inadequate reporting, but because physically non-equivalent measurement configurations were treated as comparable through normalization. The current OoC impedance landscape shows similar warning signs: rapid platform proliferation, geometry-dependent measurements, and debates over representation rather than equivalence. The lesson from earlier bioimpedance development is not that standardization is the wrong path, but that physics must precede metrics. Without physically grounded interpretation, well-documented impedance data risk remaining fundamentally incompatible — table values without practical meaning. Comparability starts with physical equivalence, not consistent units.

## TEER is not wrong, but needs reframing

Single-frequency transepithelial electrical resistance (TEER) measurements are a widely adopted tool in cellular assays. They remain effective for tight, planar epithelial barriers and for continuous quality control in systems where current paths are well defined and approximately homogeneous. In such contexts, TEER provides a sensitive, non-destructive indicator of barrier integrity over time.

Limitations arise when this scalar metric is applied to leaky epithelia, small-scale, complex 3D and vascularized structures. In these systems, current increasingly bypasses cellular junctions, and measured resistance is often dominated by fluid conductance, geometry, or electrode– interface effects rather than by barrier properties. The metric becomes underdetermined: identical values can arise from different combinations of junctional state, geometry and interface polarization. Single-frequency TEER loses discriminative power as geometry and architecture dominate current paths [7].

Frequency-resolved, parametric impedance analysis enables biological state changes to be distinguished from measurement artifacts by separating contributions from junctional resistance, membrane properties, and interface effects. Integrated Organ-on-Chip platforms incorporating multifrequency impedance analysis already exist, demonstrating technical feasibility in complex OoC geometries [8,9]. However, this transition must be accompanied by explicit treatment of model assumptions and uncertainty. Without this discipline, increased model complexity risks replacing oversimplified metrics with opaque ones. Success depends on whether impedance researchers are willing to expose model assumptions and uncertainty with the same rigor historically applied to experimental validation.

## Scaling threatens to outpace understanding

Impedance measurements produce dense, time-resolved datasets that quickly exceed what can be robustly interpreted using simple metrics. This creates strong pressure to apply machine-learning approaches. They offer a way to profile complex impedance signatures across large experimental datasets [10]. However, OoC impedance data are rich in non-biological signals: Temperature cycles, electrode polarization drift, media changes, and geometry-specific effects are stable, repeatable, and strongly correlated with experimental timelines. In many settings, these artifacts are more stable and statistically cleaner than biological signals, making them highly attractive to unconstrained models. As a result, AI models learn experimental artifacts rather than biological state changes, especially when trained and validated on a single platform or geometry [11]. High-throughput repetition and long experiment durations reinforce this failure mode, rendering biologically meaningless correlations statistically convincing.

This risk is amplified by policy-driven scaling, which dramatically accelerates progress while simultaneously magnifying the consequences of unresolved interpretation gaps. The recent spike in EIT data production in China demonstrates this pattern [12]. As OoC platforms move toward industrial adoption, data volumes will rapidly outpace current pilot studies. At that scale, data generation itself begins to shape conventions. Unless physical meaning is defined in advance, scaling will increase ambiguity rather than resolve it.

The question is not whether AI will be applied to OoC impedance data, but who will define the rules under which it is allowed to learn, and whether those rules are grounded in physical meaning rather than convenience. Impedance is not a black-box signal: it is governed by well-understood physical models and loses meaning when treated otherwise. Without explicit enforcement of physical meaning through geometry-aware parameters, model constraints, and uncertainty accounting, AI will simply scale what is stable, not what is biologically meaningful.

## Regulatory openness demands qualified metrics

Legislative and evaluation pathways now conditionally accept Organ-on-Chip evidence [13]. This acts less like a green light and more like a spotlight: weak metrics become visible, and dismissible, faster. Regulators are not asking whether impedance provides insight, but whether that insight can be qualified and defended as a meaningful representation of human biology within a defined Context of Use [14]. The burden shifts from demonstration to justification.

This shift exposes a structural weakness in how impedance data are currently positioned. Metrics only weakly linked to context, such as simple TEER values or platform-specific impedance signatures, are difficult to defend in regulatory discussions that emphasize mechanism, uncertainty, and comparability. Without explicit links between measured signals, physical models, and biological interpretation, impedance risks being viewed as exploratory rather than hard evidence. The interpretation gap cannot be resolved at the regulatory level. It can only be addressed upstream, through research practices that treat physical interpretability and qualification as non-negotiable design constraints.

Regulatory openness therefore represents both opportunity and filter. It creates space for impedance-based approaches only where interpretation, validation, and methodological discipline are evident. Whether impedance becomes part of the standard toolset will depend less on policy momentum than on the community’s willingness to meet these requirements head-on, rather than attempting to retrofit meaning afterward.

## Interpretation is the key

For the bioimpedance community, this defines a clear responsibility. If impedance is to play a role in Organ-on-Chip workflows, it must evolve from a convenient monitoring signal into a qualified measurement framework. This evolution will not be driven by improved instrumentation alone, nor by increased data volume, but by the deliberate articulation of physical meaning: explicit definition of Context of Use, geometry-aware interpretation, systematic uncertainty characterization, and transparent linking of electrical impedance to biological state.

Meeting these requirements is demanding, but it also creates opportunity. As Organ-on-Chip platforms scale and experimental complexity increases, few modalities are as well suited to continuous monitoring as impedance. At the same time, automated and AI-based interpretation will inevitably become part of these workflows. While such approaches amplify existing risks, they also offer a path forward, provided physical models are treated as guiding constraints rather than optional add-ons. Industry will be essential in translating these foundations into robust, regulatory-facing workflows, but neither scale nor automation can recover physical meaning that was never rigorously defined.

These implications directly apply to how impedance studies are designed, interpreted, reviewed, and published today. The criteria for what constitutes a meaningful impedance result are shifting, whether explicitly acknowledged or not [15]. Sensitivity without interpretability, scale without physical equivalence, and data without uncertainty assessment will increasingly fail to meet that bar.

At scale, Organ-on-Chip systems will not tolerate ambiguous signals. If system-aware, physically grounded interpretation remains secondary, impedance will be confined to supportive monitoring - valuable, but ultimately peripheral. If, instead, interpretability, physical equivalence, and qualification are treated as foundational design constraints, bioimpedance will help define how biological relevance, comparability, and evidence are established. Where the field moves from here is no longer a question of technical possibility, but of collective intent.
